# Examination Approach to the Dizzy and Swaying Patient

**DOI:** 10.21315/mjms2020.27.6.9

**Published:** 2020-12-29

**Authors:** Nur Nazleen Said Mogutham, Jafri Malin Abdullah, Zamzuri Idris, Abdul Rahman Izaini Ghani, Sanihah Abdul Halim, Jonathan Joseph J Naesarajoo, Lin-Wei Ooi, Mohamad Muhaimin Abdullah, Aiman Ashraf Ahmad Sukari

**Affiliations:** 1 Department of Neurosciences, School of Medical Sciences, Universiti Sains Malaysia, Kelantan, Malaysia; 2 Center for Neuroscience Services and Research, Universiti Sains Malaysia, Kelantan, Malaysia; 3 Brain and Behaviour Cluster, School of Medical Sciences, Universiti Sains Malaysia, Kelantan, Malaysia; 4 Unit of Neurology, Department of Medicine, School of Medical Sciences, Universiti Sains Malaysia, Kubang Kerian, Kelantan, Malaysia

**Keywords:** neurology, dizziness, nystagmus, vertigo, gait

## Abstract

**Background:**

Dizziness is a common presenting complaint among patients in Malaysia. It is a vague term which could be associated with vertigo, imbalance, ataxia or syncope. In order to deal with this overwhelming complaint, a detailed history-taking is essential in confirming aetiology of disease and this should be followed by a meticulous clinical examination. The purpose of the video manuscript it to provide a step-by-step approach to a dizzy and swaying patient, specially catered for Malaysian medical students and trainees.

**Methods:**

A series of videos were shot, which involved the eye, ear, vestibular system, cerebellar, proprioceptive sense and gait examination. These videos, conducted in Universiti Sains Malaysia (USM) School of Medical Sciences, will be first in Malaysia and will highlight the proper technique and rapport with patients and essential points of each examination. There will be summary at the end of each examination on how to report findings which is a common weakness among students.

**Conclusion:**

We hope that students and junior doctors could be apply these methods in their daily assessment of dizzy patients and ultimately, reach an accurate diagnosis.

## Introduction

Dizzy and swaying patient is commonly encountered by clinical practitioners on a regular basis. It is an archetypal symptom that can occur due to any dysfunction in any system of the body. This umbrella term could encompass life-threatening conditions that need urgent intervention, or it could lead to a benign condition. The first important step is history taking in which extra effort is needed to probe the true meaning of dizziness and distinguishing between central or peripheral causes. Next would be a systemic clinical examination to confirm the diagnosis. The key element is inspection followed by simple bed-side tests (1).

**Figure 1:**
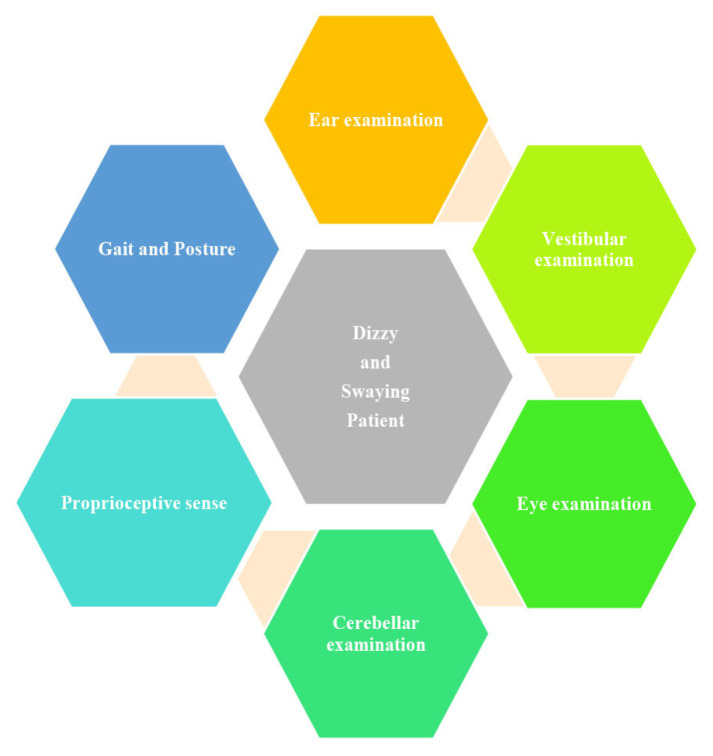
A schematic diagram of examination systems involved in the approach of the dizzy and swaying patient (2).

This systemic approach in examining a dizzy and swaying patient involves six examination systems described in Figure 1 (2). These examinations will singularly and collectively aid in differentiating between central versus peripheral cause of dizziness.

Before we begin, it should be reminded that all examinations should begin with hand washing, confirmation of patient’s identity, an explanation of the steps involved and consent with adequate exposure to preserve their dignity.

## General Inspection

By standing at the end of the bed, make it a point to make a gross assessment of the patient and their surroundings. Be vigilant on picking up small signs that may indicate the severity of the dizziness and its impact on the patient. For example, the patient may keep their eyes closed due to spinning-like sensation of their surroundings. Observe for any facial asymmetry, which could be an indication of peripheral nerve involvement or a cerebrovascular event.

Observe the patient’s head and body posture, which could show mild tilt or turn of head or body (4).

## Vital Signs and Systemic Examination

Prior to a full neurological examination, a full cardiorespiratory assessment should be conducted to rule out the cause of dizziness. Lying and standing blood pressure should be measured to rule out orthostatic hypotension and blood sugar for any hypoglycaemic events (3).

## Ear Examination

- Ask the patient if they have any hearing impairment, any pain in their ears or any discharge.
- Inspection: a head-worn light source, leaving the hands free. Look out for findings as shown in Table 1 (2, 3).
- Basic hearing assessment:

### Whispering test (5, 6)

Test one ear at a time. Block opposite ear: press on tragus/produce blocking white noise in a circular motion.

Position yourself approximately 60 cm from the patient’s ear and then whisper a number or word in a series of three.

Ask the patient to repeat the number or word back to you.

i) If they get two-thirds or more correct, then their hearing level is 12 db or better.
ii) If there is no response, use a conversational voice (48 db or worse) or loud voice (76 db or worse).
iii) If there is no response, you can move closer and repeat the test at 15 cm distance. Here, the thresholds are 34 db for whisper and 56 db for conversational voice.

Assess the other ear in the same way.

IV) Tuning fork tests

*Use tuning fork of 256 Hz

i) Rinne’s test (2–3, 7)

Instructions:

Explain to the patient that this instrument will produce vibrations and it will be placed behind the ear. Inform the patient once the vibrations have disappeared. Then place prongs by side of the ear. Ask which one is louder?

Hit prongs with tendon hammer, place on the mastoid process (bone conduction), and then in front of the ear (air conduction).

Ask the patient in which position the sound is louder.

ii) Weber’s test (2, 7)

Instructions:

Place same tuning fork at centre or middle of forehead/vertex.

Ask the patient which side is louder.

Summarise the findings and correlate with Table 2 (2, 7).

V) Otoscopy examination (8)

Instructions:

Assemble the otoscope and make sure it is functioning well.

Explain to the patient that you will be pulling their asymptomatic ear gently back and upwards to straighten the ear canal. The insertion of the otoscope would be slightly uncomfortable but not painful.

For the right ear, hold the otoscope in your right hand and rest your little finger on the patient’s cheek for stability.

**Figure 2:**
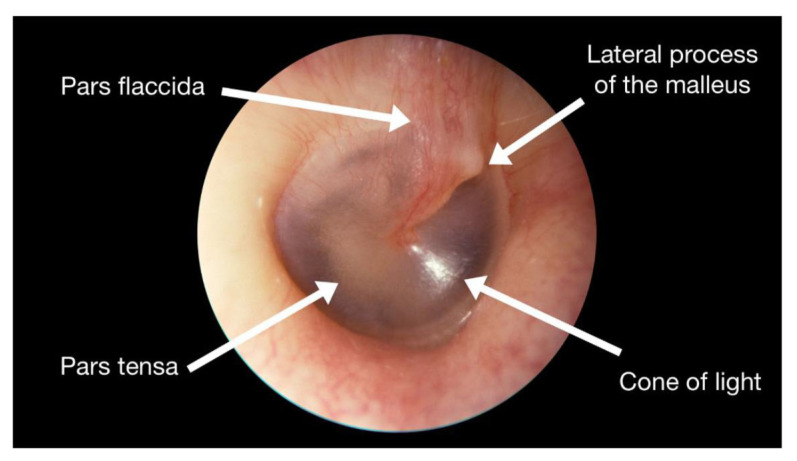
Image of normal tympanic membrane (9)

Insert the otoscope tip until the tympanic membrane is on your visual. Figure 2 (9) shows the appearance of a normal right tympanic membrane.

VI) Conclusion

Inform the patient that the examination is now complete and thank them.

Summarise and report the findings.

To complete the examination, you would like to perform a full cranial nerve examination and other examinations involved to assess a dizzy and swaying patient.

Here is a step-by-step video on ear examination.

Video link: https://youtu.be/0uAtowDHLus

## Eye Examination

I) Ask the patient if they use any visual aids, for any visual abnormalities, pain or abnormal eye movements?
II) Inspection: look for any local eye signs such as swelling, proptosis, redness or ptosis.
III) Fixation

Ask the patient to look at the fixation target (primary position → eccentric gaze → return to the primary position.

What are we looking for? → Continuous/intermittent oscillations.

Assess nystagmus by referring to Table 3 (10).

*If there is a complaint of oscillopsia but no clinical findings, repeat under slit-lamp examination.

IV) Eye movements (8, 10)
i) Testing range of movement

Purpose:

To determine the limitation of movement in one or both eyes.

Instructions:

Test the patient’s visual acuity by checking if they can visualise a red pin.

**Figure 3:**
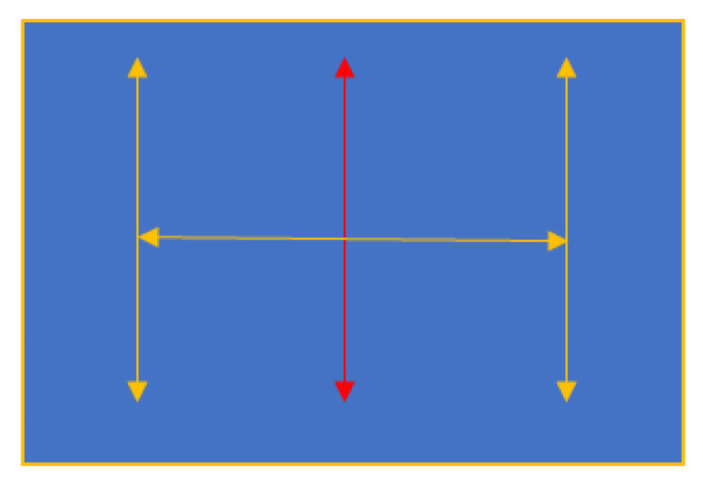
Testing six cardinal position for extraocular movements

Ask the patient to follow the target from the primary position into each of six cardinal positions with both eyes open (Figure 3).

If there appears to be a limitation of movement on one eye, reassess that movement by covering the other eye.

**Note**
- Limitation of eye movement can be caused by weak agonist & tight antagonist or vice versa
- Muscle weakness → improve with monocular assessment
- Restrictive process → same for monocular/binocular

ii) Testing pursuit movement

Purpose:

To assess how well the patient can follow a moving target (normal speed 30°/sec).

Instructions:

Ask the patient to follow a target in the horizontal and vertical planes.

Assess the quality of movement (delay?)

**Notes**
- Normal: smooth, no breaks/saccades
- Cerebellar lesions: pursuit movement may break down and become cogwheel/saccadic (series of catch-up saccades)

iii) Testing saccadic movement (10)

Purpose:

To assess how rapidly and accurately the patient can fixate on an eccentric target.

Instructions:

Hold two targets in front and on either side of the patient’s head (18 inch apart) such that the patient will make approximately 20°–30° movements from the primary position (horizontal plane).

Ask the patient to alternate between the two targets (gap of few seconds) as quickly as possible.

Repeat for vertical plane.

Assess the quality of movements for (Table 4):

i) Speed of initiation
ii) Velocity
iii) Accuracy (overshooting/undershooting)

**Notes**

Saccadic accuracy depends on the connections in the dorsal part of the cerebellar vermis and the fastigial nuclei.

- Allow only short delay between the commands (maybe prolonged in Parkinson’s disease)
- If velocity is slow → brain, nerve or muscle problem
- Undershoot of target (hypometric)
- Overshoot of target (hypermetric)
  *inaccurate in brainstem/cerebellar disease

iv) Testing convergence

Purpose:

To assess how well the patient can follow a target moving in depth; need to test if:

i) Complaint of double vision for a near object.
ii) Acquired exotropia with limited adduction on smooth pursuit testing.

Instructions:

Ask the patient to look at an accommodative target (letter/number) about 30 cm away, held perpendicular to their nose.

Move this target slowly towards the bridge of their nose, urge the patient to ‘keep it single for as long as you can’.

Observe how far the eyes adduct towards each other.

Measure the distance from the eyes at which the patient says the target becomes double (near the point of convergence - NPC)

*normally around 10 cm for all ages

v) Testing vestibulo-ocular reflex (VOR)

Purpose:

To assess how well the patient can maintain fixation during brief head or body movement.

When to perform?

i) Bilateral partial/total ophthalmoplegia (including horizontal/vertical gaze palsies)
ii) Patient complains of oscillopsia (spontaneous/upon walking) but no nystagmus present (loss of VOR in subtle or early cerebellar or brainstem disease)

Instructions:

First, ask the patient if they have any neck problems (if they do use a swivel chair instead).

To test horizontal VOR, ask the patient to keep looking at your nose, then gently but rapidly rotate their head from side to side.

To test vertical VOR, ask the patient to keep looking at your nose and tilt their head forwards and backwards.

**Notes**
- Normal response: patient’s eyes remain fixed on your nose despite the rapid movement
- Abnormal response: patient’s eye movements to lag behind their head

vi) Testing optokinetic nystagmus (OKN)

Purpose:

To assess how well the patient can maintain fixation during sustained head/body movement (rotation/translation).

*May help localise the site of lesion causing homonymous hemianopia.

Instructions:

Use OKN strip: a long strip of fabric with a repetitive stripe or figure pattern that is moved in front of the patient’s eyes.

Or use the OKN drum.

vii) Skew deviation

Vertical misalignment of the eyes is the hallmark of an imbalance in the tonic levels of activity underlying otolith-ocular reflexes.

- Often complain of vertical diplopia, sometimes with the illusion of tilt of the visual world, and the head may also be tilted.

Perform the ocular cover test.

Instructions:

Examiner moves a cover from one of the patient’s eyes to the other while watching for vertical corrective eye movement when the cover is switched.

V) Conclusion

Inform the patient that the examination is now complete and thank them.

Summarise and report findings.

To complete the examination, you would like to perform a full cranial nerve examination and other examinations involved in the assessment of a dizzy and swaying patient.

Here is a step-by-step video on eye examination.

Video link: https://youtu.be/a2cC_X4Z0XM

## Vestibular System Examination

Four clinical tests are useful tools in evaluating vestibular function:

i) Head impulse test
ii) Romberg test
iii) Fukuda-Unterberger test
iv) Dix-Hallpike manoeuvre
v) Hennebert’s test
i) Head impulse test (1, 11)

Purpose:

Test the VOR – to differentiate between central and peripheral cause (sensitive and specific to detect unilateral hypofunction of the peripheral vestibular system).

In patients with acute vestibulopathy, when the head is turned toward the affected side, there will be a delay in vestibular adjustment (manifest as a brief and fixed gaze toward the affected side followed by a corrective saccadic eye movement back to the centre).

Instructions:

Explain the test to the patient clearly.

Get the patient to sit in front of the examiner and hold the patient’s head steady in the midline.

Instruct the patient to maintain gaze on the nose of the examiner.

Turn the patient’s head quickly about 10°–15° to one side and observes the ability of the patient to keep their eyes locked on the examiner’s nose.

ii) Romberg test (7, 12)

Purpose:

Assesses the integrity of peripheral proprioception, cerebellar, and vestibular functions.

Instructions:

Assure the patient that you will be ready to catch them if they experience some dizziness.

Ask the patient to maintain their balance with their both feet placed closely together.

Get the patient to close their eyes.

**Positive**: when the patient can maintain their balance with both feet placed close together with visual input, but not when their eyes are closed.

iii) Fukuda-Unterberger test (8, 12)

Purpose:

To assess labyrinthine function via vestibulospinal reflexes.

Instructions:

Assure the patient that you will be ready to catch them if they experience some dizziness.

Ask the patient to march on the spot with their eyes closed.

**Positive**: when the patient deviates from the midline, usually toward the side with a relatively lower vestibular activity.

iv) Dix-Hallpike manoeuvre (12, 13)

Should be performed if the history suggestive of benign paroxysmal positional vertigo (BPPV) or if the nystagmus is inducible.

Purpose:

Used to diagnose the posterior semi-circular canal variant of BPPV.

Instructions:

Explain to the patient the steps involved in the Dix-Hallpike manoeuvre and constant reassurance during the process can help reduce the patient’s discomfort and anxiety.

Emphasise that they need to keep their eyes open and suggest them to look at your nose.

Perform this manoeuvre first on the asymptomatic side.

With the patient sitting, the head is turned 45° to the side placing the posterior canal on that side in the sagittal plain.

The patient is then moved swiftly to the head-hanging position (head and neck are extended at least 20° below the horizontal plane).

Important to wait for at least 30 sec to observe for nystagmus.

**Notes**
*If the patient has debris moving in the posterior canal, this will lead to a very specific pattern of nystagmus: a burst of upbeat-torsional nystagmus lasting about 15 sec.
*Pure vertical nystagmus, particularly persistent downbeat nystagmus, suggesting a central lesion, usually involving the midline cerebellum.

v) Hennebert’s test (14)

Purpose: Assess the integrity of vestibular, cerebellar, and proprioception function.

Instructions:

Explain to the patient that the next test could probably induce vertigo or eye signs, reassure their safety.

Ask the patient to press into their tragus and hold.

Observe for nystagmus and vertigo.

vi) Conclusion

Inform the patient that the examination is now complete and thank them.

Summarise and report the findings.

To complete the examination, you would like to perform a full cranial nerve examination and other examinations involved in the assessment of a dizzy and swaying patient.

Here is a step-by-step video on vestibular examination.

Video link: https://youtu.be/PSfUC1T9OBY

## Cerebellar Examination

I) Ask the patient if they have any problems with imbalance or coordination?
II) Inspection (head to toe survey):
i) Head: titubation (spasmodic nodding of head), surgical scar or eyes for nystagmus
ii) Truncal ataxia
iii) Limbs: tremors, broad-based gait, veering to one side (unilateral lesions).
III) Check the eyes for diplopia, saccadic dysmetria, and nystagmus (Please refer to eye examination section and video).
IV) Speech (8)

Instructions:

Ask the patient to repeat a couple of short phrases such as ‘Baby hippopotamus’/’British Constitution’/‘*Persatuan Peladang-peladang Pulau Pinang*’.

Observe for ataxic dysarthria:

i) Slow or slurred speech due to lack of coordination
ii) Staccato speech – explosive character
iii) Scanning speech – slow and accentuate syllable by syllable and normal prosodic rhythm is lost.

Note: ataxic dysarthria is due to an injury on the left of the cerebellar hemisphere.

V) Upper limb assessment
i) Tone

*Rebound phenomenon* (15)

Purpose: Test for hypotonia and dysmetria.

Instructions:

Ask the patient to keep arms outstretched forwards with their eyes closed.

Instruct them to keep their arms steady as you lightly push their arms down (push at forearm).

Note: positive rebound phenomenon occurs when the patient’s arms overshoot when repositioning due to the inability of antagonistic muscle the check the sudden change in movement.

ii) Coordination

*Rapid alternating movements* (8)

Purpose: Test for smoothness of rapid movement.

Instructions:

Test asymptomatic side first.

Ask the patient to place their right palm on top of the left.

Next, instruct them to repeatedly flip their right hand at an increasing pace.

After about 5 sec of continual movement, ask them to do the same with their left hand.

Note: if the patient is unable to perform test smoothly and rapidly (dysdiadokinesia), it is indicative of an ipsilateral cerebellar lesion.

Finger-to-Nose Test (8)

Purpose: Test for dysmetria and intention tremor.

Instructions:

Test asymptomatic side first.

Ask the patient to touch their index finger to their nose.

Hold your own finger at an arm’s length distance away and instruct the patient to touch your index finger with theirs, before touching their nose once again.

Ask them to repeat this motion with both hands.

Note: If present, it is indicative of an ipsilateral cerebellar lesion.

VI) Lower limb assessment
i) Tone

*Pendular reflex* (8)

Purpose: Test for hypotonia, based on the number of leg swings.

Instructions:

Test the asymptomatic side first.

Test the patellar reflex (L3–L4) in both legs: Take the weight of the leg and ask the patient to relax.

Tap the patellar tendon, which is superior to the tibial tuberosity and inferior to the patella.

Note: Pendular reflexes will be slow, and the leg will continue to swing back and forth (like a pendulum). More than 4 swings is pathological.

ii) Coordination

*Heel-shin test* (8)

Purpose: Test for dysmetria.

Instructions:

Test the asymptomatic side first.

Ask the patient to place their right heel on their left knee.

Next, instruct them to move their right heel down to their left ankle, and then lift their foot in the air such that their toes touch your hand.

Then ask the patient to repeat this motion as rapidly as possible.

After about 5 sec of continuous movement, ask them to do the same using the left heel instead.

iii) Conclusion

Inform the patient that the examination is now complete and thank them.

Summarise and report the findings.

To complete the examination, you would like to perform a full cranial nerve examination and other examinations involved in the assessment of a dizzy and swaying patient.

Here is a step-by-step video on cerebellar examination.

Video link: https://youtu.be/SWgi5w-eFmU

## Proprioceptive Sense Examination

I) Ask the patient if they have any imbalance or reduced sensation.
II) General inspection.
III) Vibration sense (8)

*Use 128 Hz tuning fork

Purpose: Assessment of the integrity of the dorsal column-medial lemniscus pathway.

Instructions:

Ensure the patient understands that they must feel the vibration, by striking the fork and placing on sternum/forehead as a reference.

Begin on the asymptomatic side.

Ask the patient to close their eyes.

Place tuning fork at the most distal bony prominence of upper/lower limb and ask if they can feel the vibration.

Repeat on the other side.

IV) Joint position sense (8)

With the patient’s eyes open, show them what you are going to do.

Hold the distal phalanx between the two fingers (ensure you are not holding nail/pulp of finger).

Ensuring that your fingers are at 90° to the intended direction of movement, move the digit, illustrating which is up and which is down.

Ask the patient to close their eyes and repeat the movement and get them to guess it.

Test distal joints. If abnormal, then test more on proximal joints.

Here is a step-by-step video on proprioceptive sense examination.

Video link: https://youtu.be/a_og9chUKZY

## Gait Examination (1, 11)

I) Ask the patient if they have any imbalance, trouble in walking or frequent falls.

Purpose:

Impaired gait and balance can accompany dizziness of any cause but as a rule, severe gait impairment suggests a neurologic disorder.

Instructions:

Ask the patient to walk across the room (at least 5 m).

Ask the patient to walk as if on a tight rope (tandem gait).

Here is a step-by-step video on gait examination.

Video link: https://youtu.be/9YJ74dUpUWw

We hope that systemic examination in the approach of deciphering the presenting complaint dizziness will play a role in finalising a clinical diagnosis. Remember that a good history is vital and complimented with these steps, will help us differentiate a sinister condition from a benign one. Table 5 summarises all the examinations involved and the interpretation of positive results.

## Figures and Tables

**Table 1:** Summary of abnormalities to look out for during ear inspection (2, 3)

Examination of	Inspect for
Auricle (pinna)	signs of inflammation, trauma, surgical scars, or haematoma following a blow to the ear, and also for congenital deformities, vesicles on pinna
External auditory meatus	Use speculum of otoscope, direct around circumference of outer ear canal. Look for: – debris – foreign bodies – inflammation/infection (for example otitis externa, vesicles in Ramsey-Hunt syndrome – defects of posterior/anterior wall
Tympanic membrane and middle ear	Wax Identify: – perforations – colour of eardrum – light reflex lost if membrane thickened/tympanosclerosis – position of membrane: retraction/bulging outwards – evidence of infection → otitis media

**Table 2:** Tuning fork test findings and its interpretations (2, 7)

Tuning for tests		Interpretation
**Rinne’s test**	**Positive**	if air conduction (AC) > bone conduction (BC) — that is, the sound in front of the ear is reported as louder: – indicates normal hearing – or an ear with a sensorineural hearing loss.
	**Negative**	If BC > AC (sound behind ear is quieter) – indicates significant conductive component of hearing loss > 15 dBHL
**Weber’s test**	**Central (equal both sides)**	Normal hearing
	Lateralising weber	Identifies the side of the better hearing cochlear

**Table 3:** Elements involved in assessment of nystagmus (10)

Elements of Nystagmus	How to Report
Influence of eye position	Is it present in primary position/only eccentric gaze? Does the plane vary with direction of gaze? Does intensity vary with direction of gaze/with convergence? Is there a position where: Intensity is the least (null zone)? Direction of jerk nystagmus reverses (neutral zone)?
Waveform	Pendular/sinusoidal Jerk (slow movement away from fixation & fast corrective movement in opposite direction) Mixed
Plane	Horizontal/vertical/torsional/mixed
Direction	*direction of fast phase = direction of jerk nystagmus *if changes direction after several minutes → periodic alternating nystagmus
Conjugacy	*Conjugate*: jerk/pendular phases of both eyes in same direction *Disconjugate*: fast & slow phases are in different direction
Influence of fogging/occlusion	Is intensity increased by fogging Does the nystagmus only occur or change direction when one eye is occluded?
Influence of nystagmus on eye movements	Does nystagmus break up pursuit eye movements?

**Table 4:** Elements involved in assessment of saccades (10)

Elements of Saccadic Movements	How to Report
Plane	Horizontal/vertical/oblique
Amplitude	Small (< 5°) Large (> 5°)
Frequency	High versus low
Duration	Intermittent (bursts) Continuous (oscillations)

**Table 5:** Summary of relevant clinical findings in full examination of dizzy and swaying patient and its interpretation (2, 10, 16–18)

Type of examination	Clinical tests	Clinical findings	Relevance	
**Ear Examination**	Hearing assessment	New hearing loss	Acute ischemia of labyrinth or brainstem Ménière’s disease Acoustic neuroma	
	Rinne’s & Weber’s test	Conductive hearing loss	Middle or external ear disease, may cause dizziness if tympanic membrane is breached	
		Sensorineural hearing loss	Vestibular nerve involvement	
	Hennebert’s test (Fistula sign)	nystagmus in association with vertigo (nystagmus towards affected ear)	indicates bony destruction in the inner ear e.g. cholesteatoma, perilymphatic fistula.	
**Eye Examination**	Eye position	Squint Nystagmus	May cause double vision	
	Cover/Uncover test	Skew deviation	Suggestive of central lesion	
			*Peripheral lesion*	*Central lesion*
	H test	Smooth pursuit	Smooth	Broken/jerky
	Nystagmus	Direction	Unidirectional (mixed pattern)	Uni- or bi-directional, purely one direction
		Suppress with visual fixation	Yes	No
	Positional nystagmus	Fatigability?	Yes	No
	Saccades	Hypo/hypermetria	Tendency to occur in cerebellar pathology	
	Optokinetic nystagmus (OKN)	Unable to maintain fixation	Parietal lesions may have reduced ipsilateral OKN response.	
**Vestibular System Examination**	Head impulse test	Unable to maintain fixation upon turning of head	Peripheral – impaired Central - intact	
	Romberg’s test	Instability or tendency to fall	Indicative of deficit in dorsal column-medial lemniscus pathway.	
	Fukuda-Unterberger test	Drift from midline	Turn towards lesion with lower vestibular activity.	
	Dix-Hallpike manoeuvre	Vertical upwards-rotational nystagmus beating towards the ground	Highly suggestive of posterior Benign Paroxysmal Positional Vertigo (BPPV).	
**Cerebellar Examination**	Nystagmus	Down beat	lesion at floccular–parafloccular (tonsil) complex and the nodulus craniocervical anomalies drug intoxication	
		Periodic alternating nystagmus	nodulus and uvula	
		Upbeat nystagmus	Lesions in medulla Superior cerebellar peduncle	
		Gaze-evoked nystagmus	Involving vestibulocerebellum	
	All other steps		Localizes to ipsilateral cerebellum	
**Proprioceptive Senses**	Vibration sense	Impaired	Suggestive of involvement of dorsal-column-medial-lemniscus tract.	
	Joint position sense	Impaired		
**GAIT Assessment**	Normal and tandem gait	Look for imbalance/swaying/ataxic gait	*poor localising sign but confirms neurological disorder. Peripheral disorders cause imbalance but patient may walk unassisted. Broad based gait suggest ataxia.	
